# Differential Expression of VGLUT2 in Mouse Mesopontine Cholinergic Neurons

**DOI:** 10.1523/ENEURO.0161-19.2019

**Published:** 2019-08-12

**Authors:** Thomas Steinkellner, Ji Hoon Yoo, Thomas S. Hnasko

**Affiliations:** 1 Department of Neurosciences, University of California, San Diego, La Jolla, California 92093; 2 Veterans Affairs San Diego Healthcare System, Research Service, San Diego, California 92161

**Keywords:** acetylcholine, cholinergic neurons, co-release, genetic tracing, glutamate, vesicular glutamate transporter

## Abstract

Vesicular glutamate transporters (VGLUTs) mediate the synaptic uptake of glutamate from the cytosol into synaptic vesicles and are considered unambiguous neurochemical markers of glutamate neurons. However, many neurons not classically thought of as glutamatergic also express a VGLUT and co-release glutamate. Using a genetic fate-mapping strategy we found that most cholinergic neurons in the mouse mesopontine tegmentum express VGLUT2 at some point during development, including the pedunculopontine tegmental nucleus (PPTg), laterodorsal tegmental nucleus, and parabigeminal nucleus (PBG), but not the oculomotor nucleus. In contrast, very few of these cholinergic neurons displayed evidence of vesicular GABA transporter expression. Using multiplex fluorescent *in situ* hybridization, we determined that only PBG cholinergic neurons are also predominantly positive for VGLUT2 mRNA in the adult, with only small numbers of PPTg cholinergic neurons overlapping with VGLUT2 mRNA. Using Cre-dependent viral vectors we confirm these *in situ* hybridization data, and demonstrate projection patterns of cholinergic and glutamatergic populations. These results demonstrate that most mesopontine cholinergic neurons may transiently express VGLUT2, but that a large majority of PBG neurons retain VGLUT2 expression throughout adulthood, and support a growing body of literature indicating that distinct cholinergic populations have differing potential for GABA or glutamate co-release.

## Significance Statement

Many cholinergic neurons can engage in fast excitatory or inhibitory neurotransmission via glutamate or GABA co-release; however this has not been carefully assessed throughout mesopontine cholinergic cell groups. We show that VGAT colocalization is uniformly very low, suggesting minimal potential for GABA co-release from mesopontine cholinergic cells. On the other hand, VGLUT2 expression is more abundant and variable across these populations. VGLUT2 may be transiently expressed widely in these cells during development, and its expression persists in PBG cholinergic neurons of the adult. Our data imply a possible developmentally dynamic role for VGLUT2 in mesopontine cholinergic neurons, as well as a role for glutamate/acetylcholine co-release in visuomotor control driven by PBG.

## Introduction

Chemical neurotransmission is the main mode of communication for interneuronal signaling, underlying essentially all basic physiologic processes such as respiration or movement, as well as complex cognitive functions such as learning and memory. The bulk of synaptic neurotransmission is mediated by a surprisingly small group of classical transmitters, which include the amino acid transmitters, monoamines, purines, and acetylcholine (ACh). A defining feature of these transmitters is their ability to be synthesized, packaged and recycled at the nerve terminal, through a process dependent on vesicular transporters that catalyze filling of synaptic vesicles. However, we now know that multiple classical transmitters can be released from the same neuron (Sulzer and Rayport, 2000; Trudeau and El Mestikawy, 2018). For instance, a subset of midbrain dopamine neurons expresses vesicular transporters for dopamine (vesicular monoamine transporter) and glutamate [vesicular glutamate transporter (VGLUT2)], and can release both dopamine and glutamate from synaptic terminals (Chuhma et al., 2004; Hnasko et al., 2010; Stuber et al., 2010). Similarly, there are subsets of neurons that cotransmit ACh and glutamate (Gras et al., 2008; Ren et al., 2011; Frahm et al., 2015; Sakae et al., 2015), ACh and GABA (Saunders et al., 2015a,b); serotonin and glutamate (Amilhon et al., 2010; Voisin et al., 2016; Sengupta et al., 2017); and even glutamate and GABA (Root et al., 2014; Shabel et al., 2014; Yoo et al., 2016).

Subpopulations of cholinergic neurons in the brain and spinal cord were among the first neuronal populations shown to express VGLUTs enabling cotransmission of glutamate with ACh (Gras et al., 2002; Herzog et al., 2004; Kraus et al., 2004; Nishimaru et al., 2005). For instance, some spinal cholinergic motor neurons express VGLUT2 and co-release glutamate to excite inhibitory Renshaw cells (Nishimaru et al., 2005), whereas striatal cholinergic interneurons have been shown to express VGLUT3 and co-release glutamate in the striatum (Gras et al., 2008; Nelson et al., 2014). Similarly, cholinergic neurons in the medial habenula (MHb) express VGLUT1 and co-release ACh and glutamate in the interpeduncular nucleus (IPN; Ren et al., 2011; Frahm et al., 2015). More recently, it was demonstrated that subpopulations of cholinergic forebrain projection and cortical interneurons can co-release GABA (Saunders et al., 2015a,b; Granger et al., 2018). These findings suggest that many central cholinergic subpopulations can cotransmit other classical neurotransmitters besides ACh. Although some reports suggest the potential for co-release from midbrain and hindbrain cholinergic projection neurons, it has not yet been systematically investigated (Clements and Beitz, 1991; Lavoie and Parent, 1994; Ford et al., 1995; Wang and Morales, 2009; Xiao et al., 2016; Estakhr et al., 2017; Aitta-Aho et al., 2018).

Here, we provide genetic and histochemical evidence that the majority of pedunculopontine tegmental nucleus (PPTg), laterodorsal tegmental nucleus (LDTg), and parabigeminal nucleus (PBG), but not oculomotor (3N) cholinergic neurons in the mouse mesopontine tegmentum expressed a fluorescent reporter indicative of VGLUT2 promoter activity. Hence, VGLUT2 was likely expressed at least at some point during neuronal development in these cells. However, in adult mice we found that only PBG cholinergic neurons were predominantly co-positive for VGLUT2 mRNA, whereas relatively few PPTg cholinergic neurons continued to express VGLUT2 in the adult. In contrast, there was little to no evidence for expression of the vesicular GABA transporter (VGAT) in these subpopulations.

## Materials and Methods

### Mice

Mice were used in accordance with protocols approved by the Institutional Animal Care and Use Committee. Mice expressing Cre under control of VGLUT2 (*Slc17a6^IRESCr^* ^e^; 016963), VGAT (*Slc32a1^IRESCre^*; 016962), and ChAT (*Chat^IRESCre^*; 006410) regulatory elements were purchased from The Jackson Laboratory and bred in house. For fate-mapping experiments, we crossed either VGLUT2-Cre or VGAT-Cre animals with mice expressing a floxed stop cassette blocking expression of a zsGreen reporter (B6.Cg-*Gt(ROSA)^26Sortm6(CAG-ZsGreen1)Hze^/*J; 007906). VGLUT2-Cre and zsGreen reporter mice were of C57BL/6 background, and VGAT-Cre and ChAT-Cre of mixed C57BL/6 ×Sv129 background. Both sexes were used; mice were group-housed on a 12 h light/dark cycle, with food and water available *ad libitum*.

### Stereotactic surgery

Mice (>8 weeks) were anesthetized with isoflurane (1–2%) and placed into a stereotaxic frame (David Kopf Instruments). For microinfusion of virus (150–300 nl), a custom-made 30-gauge stainless steel injector was used to infuse virus unilaterally at 100 nl/min using a micropump (WPI UltaMicroPump). Injector was kept in place for 10 min before slowly retracting to allow for diffusion and to minimize backflow. Following infusion, mice were allowed to recover for at least 21 d before performing histology. We used either AAV1-EF1α - ChR2(H134R)-mCherry (for VGLUT2-Cre PPTg injections) or AAV5-EF1α-ChR2(H134R):eYFP (for ChAT-Cre PPTg and PBG as well as VGLUT2-Cre PBG injections). Adeno-associated viruses (AAVs) were obtained from the UNC Viral Vector Core. The following injection coordinates (in mm from bregma) were used: PBG: AP −4.24, ML −1.75, DV −3.25; PPTg: AP −4.48, ML −1.1, DV −3.75.

### Immunohistochemistry

Mice were anesthetized with a lethal dose of pentobarbital (100 mg/kg, i.p.), and transcardially perfused with ice-cold PBS followed by 4% paraformaldehyde (PFA). Brains were postfixed in 4% PFA overnight at 4°C, transferred to 30% sucrose for 48–72 h for cryoprotection and frozen in isopentane chilled on dry ice. Brains were coronally cut at 30 μm using a cryostat (CM3050S, Leica) and collected in PBS containing 0.01% sodium azide. Free-floating sections were washed three times for 5 min in PBS, blocked for 1 h in PBS containing 5% normal donkey serum and 0.3% Triton X-100 (blocking buffer) followed by incubation with one or more of the following antibodies in blocking buffer overnight at room temperature: goat anti-ChAT (1:500; AB144P, Millipore), rabbit anti-GFP (1:2000; AB11122, Invitrogen) or chicken anti-GFP (1:2000; AB10262, Invitrogen) to amplify Channelrhodopsin-2:eYFP (ChR2:eYFP), rabbit anti-DsRed (1:2000; AB632496, Clontech) to amplify ChR2:mCherry, rabbit anti-VAChT (1:1000; AB139103, Synaptic Systems), and guinea pig anti-VGLUT2 (1:2000; AB2251, Millipore). The following day, sections were washed three times (15 min) in PBS and incubated with secondary antibodies in blocking buffer for 2 h at room temperature. Secondary antibodies were from Jackson ImmunoResearch and conjugated to either AlexaFluor 488, AlexaFluor 594, or AlexaFluor 647 (5 μg/ml). After secondary antibody incubation, sections were rinsed three times in PBS for 15 min, mounted onto glass slides (Fisher Scientific) and coverslipped using Fluoromount-G mounting medium (Southern Biotech) ± DAPI (0.5 μg/ml). Images were captured using a Zeiss Axio Observer Epifluorescence microscope with or without ApoTome structured illumination or a Leica SP5 confocal microscope.

### *In situ* hybridization

Mice (C57BL/6j wild-type) were anesthetized with pentobarbital and killed by decapitation. Brains were extracted rapidly, frozen in chilled isopentane and stored at −80°C. Sections were serially cut (20 μm) on a cryostat and mounted directly onto glass slides (Fisher Scientific). Slides were stored at −80°C until starting the multiplex fluorescent RNAscope assay (Advanced Cell Diagnostics). Briefly, sections were fixed with 4% PFA for 15 min at 4°C followed by dehydration in increasing ethanol concentrations and protease IV treatment. RNA hybridization probes included antisense probes against genes encoding mouse VGLUT2 (*Slc17a6*; 319171-C1), ChAT (*Chat*; 408731-C2), and VGAT (*Slc32a1*; 319191-C3). Slides were counterstained with DAPI and coverslipped using Fluoromount-G mounting medium. Images were taken at 20× magnification using a Zeiss Axio Observer epifluorescence microscope.

### Cell counting

Sections covering the rostrocaudal extents of the PPTg (bregma −4.2 to −4.9), PBG (bregma −4.2 to −4.6), 3N (bregma −3.8 to −4.2) or LDTg (bregma −4.8 to −5.5) were collected and stained as described. All images were acquired using a Zeiss epifluorescence microscope (Axio Observer) or Leica SP5 confocal microscope. We counted ChAT-positive cholinergic neurons, and colocalization with zsGreen for fate-mapping experiments using ImageJ (NIH) or Zeiss Zen software. For viral tracing experiments, ChR2:eYFP- or ChR2:mCherry-labeled cells in the PBG or PPTg of VGLUT2-Cre mice were counted, and colocalization with ChAT was evaluated in ImageJ. For *in situ* hybridization experiments we counted ChAT labeled cells in either the PBG, PPTg, or 3N and assessed colocalization with either VGLUT2 or VGAT mRNAs using ImageJ. For RNAscope, a cell was deemed positive for a given mRNA if at least four puncta were present in close proximity to a DAPI-labeled nucleus. For both, immunohistochemistry and RNAscope experiments, three to four sections (covering rostral to caudal extents of the respective nuclei and spaced ∼100–150 μm) from at least three to six animals were counted.

### Statistics

GraphPad Prism (GraphPad Software) was used to analyze cell counting data and create graphs. All data are expressed as percentages after calculating mean ± SEM.

## Results

### Expression of VGLUT2 but not VGAT reporter in the majority of mesopontine cholinergic neurons

**Figure 1.**
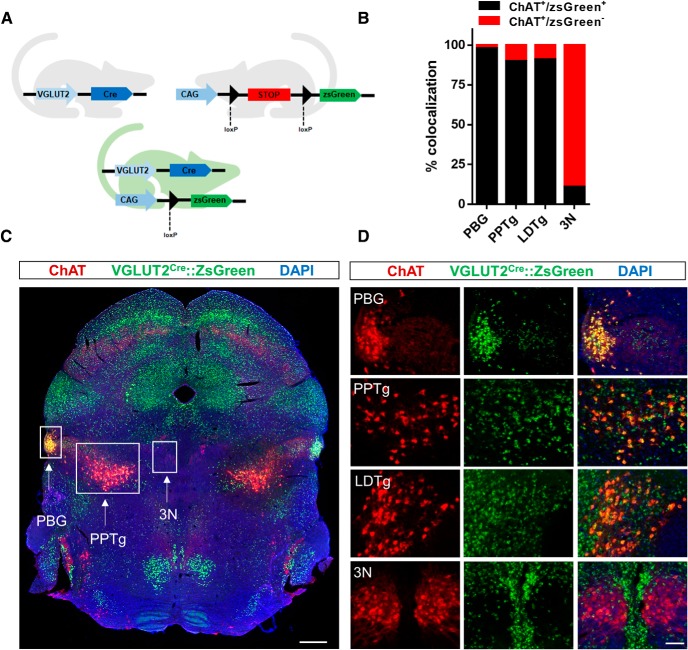
Fate-mapping of VGLUT2 expression in VGLUT2-Cre x zsGreen mice indicates that the majority of mesopontine cholinergic neurons are positive for the fluorescent reporter zsGreen. ***A***, Schematic for VGLUT2-Cre dependent expression of zsGreen. ***B***, Percentage of ChAT-positive neurons expressing zsGreen; *n* = 3–4 animals/group. ***C***, Tiled midbrain section from a VGLUT2-Cre x zsGreen mouse showing endogenous zsGreen fluorescence (green) and immunostaining for ChAT (red) as well as counterstaining with DAPI (blue). Cholinergic nuclei PBG, PPTg and 3N are denoted. Scale bar, 500 μm. ***D***, Higher-power images of PBG, PPTg, 3N, and LDTg. Note that images are not from section illustrated in panel ***C***. Scale bar, 100 μm.

We used a fate-mapping strategy to permanently label cells expressing VGLUT2 with a strong, primarily cell body-localized, fluorescent marker by crossing floxed-STOP zsGreen reporter mice with VGLUT2-IRES-CRE mice that express Cre recombinase under the control of *Slc17a6* (VGLUT2) gene regulatory elements (VGLUT2-Cre x zsGreen mice; Fig. 1*A*). Once VGLUT2 gene expression is active, Cre recombinase is expressed and excises the floxed stop cassette to permit expression of the zsGreen reporter, thereby allowing for permanent labeling of these cells with the fluorescent protein. Immunohistochemical processing and staining for the cholinergic marker choline acetyltransferase (ChAT) revealed that >90% of mesopontine cholinergic neurons in the PBG and PPTg coexpressed zsGreen (PBG: 98.4 ± 0.42%, *n* = 4; PPTg: 90.3 ± 0.33%, *n* = 3; Fig. 1*B*–*D*). Similarly, >90% of LDTg cholinergic neurons were zsGreen-positive, but only ∼10% of neurons in the 3N expressed zsGreen (LDTg: 90.9 ± 2.21%, *n* = 4; 3N: 10.8 ± 2.62%, *n* = 4; Fig. 1*B*–*D*) suggesting that most, but not all mesopontine cholinergic neurons had the potential for VGLUT2 expression.

**Figure 2.**
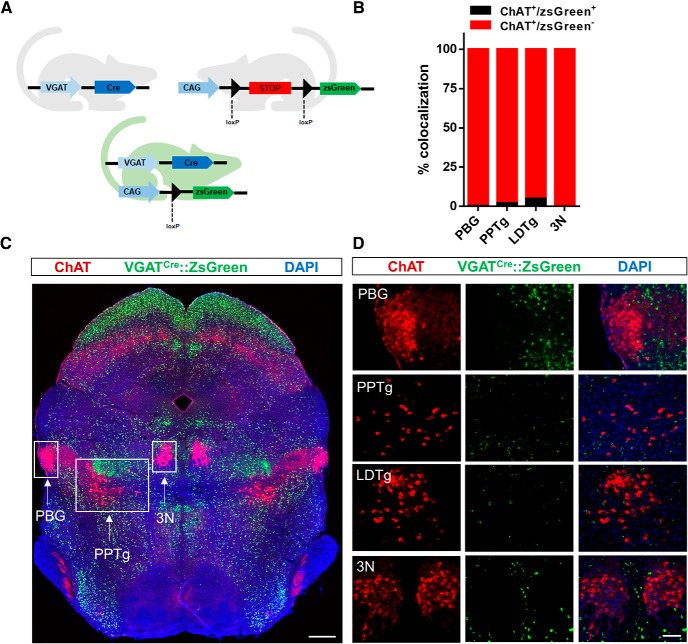
VGAT-Cre x zsGreen mice reveal that mesopontine cholinergic neurons are rarely coexpressing the VGAT fluorescent reporter zsGreen. ***A***, Schematic for VGAT-Cre dependent expression of zsGreen. ***B***, Percentage of ChAT-positive neurons expressing zsGreen; *n* = 3–4 animals/group. ***C***, Tiled midbrain section from VGAT-Cre x zsGreen mouse showing endogenous zsGreen fluorescence (green) and immunostaining for ChAT (red) and nuclear labeling with DAPI (blue). Cholinergic nuclei PBG, PPTg, and 3N are denoted. Scale bar, 500 μm. ***D***, Higher-power images of PBG, PPTg, 3N, and LDTg. Note that images are not from section illustrated in panel ***C***. Scale bar, 100 μm.

Several forebrain cholinergic subpopulations have been shown to express markers for GABA transmission such as VGAT or glutamic acid decarboxylase 2 (Saunders et al., 2015a). To determine, whether mesopontine cholinergic neurons have the potential to express VGAT, we performed a fate-mapping approach similar to that described in the prior paragraph. We crossed VGAT-Cre mice with zsGreen reporter mice to permanently label GABAergic neurons with zsGreen (Fig. 2*A*). Immunolabeling for ChAT showed that only minor fractions of cholinergic neurons in the PPTg (2.0 ± 1.53%, *n* = 3) and LDTg (4.9 ± 2.07%, *n* = 4), and virtually none of the neurons in PBG (0.3 ± 0.25, *n* = 4) or 3N (0.0 ± 0.00%, *n* = 3) were zsGreen positive (Fig. 2*B*–*D*). Together these data suggest that most cholinergic mesopontine neurons in the PPTg, LDTg, and PBG at least transiently expressed VGLUT2, but few of these cholinergic cells expressed the GABA marker VGAT.

### The majority of PBG but not PPTg cholinergic neurons express VGLUT2 mRNA in the adult

**Figure 3.**
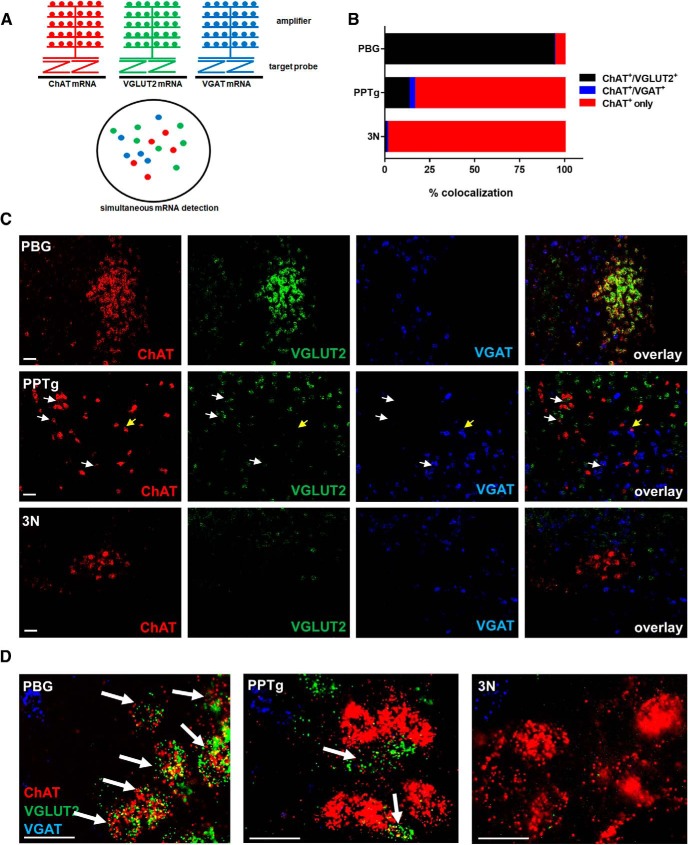
Multiplex fluorescent *in situ* hybridization reveals that most PBG but not PPTg or 3N cholinergic neurons express VGLUT2 mRNA in the adult. ***A***, Schematic of fluorescent multiplex *in situ* hybridization (RNAscope) approach using antisense probes directed against ChAT (red), VGLUT2 (green), and VGAT (blue) mRNAs. ***B***, Quantification of ChAT^+^/VGLUT2^+^ and ChAT^+^/VGAT^+^ neurons in the PBG, PPTg, and 3N; *n* = 3 animals/group. ***C***, C57BL/6 mice (10–15 weeks) were stained for ChAT (red), VGLUT2 (green), and VGAT (blue) mRNAs and counterstained with DAPI. Scale bar, 50 μm. ***D***, Magnified images of PBG, PPTg, and 3N labeled for ChAT (red), VGLUT2 (green), and VGAT (blue) mRNAs. Scale bar, 25 μm. White arrows indicate colocalization of ChAT and VGLUT2; yellow arrow indicates colocalization of ChAT and VGAT.

The advantage of the genetic approach described above is that even cells which transiently express Cre recombinase driven by either VGLUT2 or VGAT regulatory elements can be permanently labeled with the fluorescent reporter. However, reporter expression does not necessarily imply that these cells continue to express VGLUT2 or VGAT in the adult, because brief promoter activity can suffice to drive Cre expression and thus allow for permanent reporter labeling. Hence, investigating expression of VGLUT2 and VGAT in the adult is necessary to determine the at-time profile of their expression. Because the vesicular neurotransmitter transporter proteins VGLUT2 or VGAT are primarily localized to synaptic terminals, their immunolabeling cannot be reliably used to identify positive cell bodies. We therefore relied on multiplex fluorescent *in situ* hybridization (RNAscope) to identify VGLUT2 and VGAT mRNAs present in the cell bodies, and assessed their overlap with ChAT mRNA to detect potential coexpression in adult mouse brain. Interestingly, only PBG cholinergic cells showed a high rate of VGLUT2 colocalization with ChAT (93.8 ± 3.94%, *n* = 3), whereas much smaller proportions of PPTg (13.3 ± 3.28%, *n* = 3) or 3N (0.5 ± 0.51%, *n* = 3) cholinergic neurons expressed detectable VGLUT2 (Fig. 3*A*–*D*). VGAT colocalization was low in PPTg cholinergic neurons (4.0 ± 0.44%, *n* = 3) and virtually undetectable in PBG or 3N cholinergic populations (PBG: 0.1 ± 0.11%, *n* = 3; 3N: 1.0 ± 0.98%, *n* = 3; Fig. 3*A*–*D*). Note that cells negative for ChAT but positive for VGLUT2 or VGAT were present, but not quantified in this dataset.

### Viral tagging of ChAT-Cre and VGLUT2-Cre lines labels largely overlapping population of PBG neurons and projection targets

**Figure 4.**
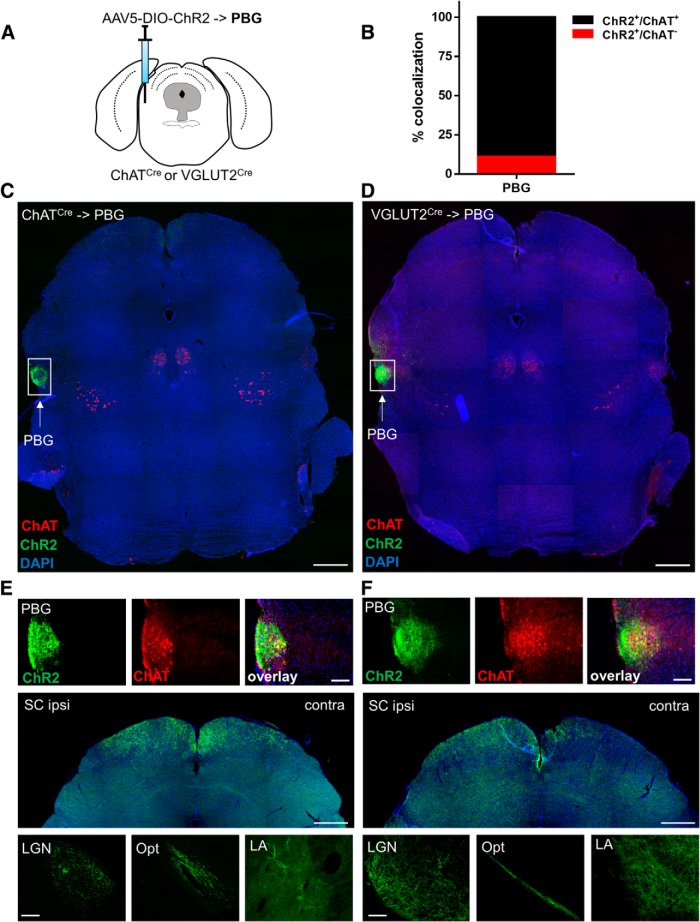
Viral tracing in PBG neurons of ChAT-Cre and VGLUT2-Cre mice. ***A***, Schematic of AAV5-DIO-ChR2:eYFP injection into the PBG of Cre lines. ***B***, Quantification of ChR2^+^ cells co-positive for ChAT counted in the PBG of VGLUT2-Cre mice; *n* = 4 animals. ***C***, ***D***, Tiled midbrain images of ChR2:eYFP (green) expression in the PBG of ChAT-Cre (***C***) and VGLUT2-Cre (***D***) mice stained for ChAT (red) and counterstained with DAPI (blue). ***E***, ***F***, Higher-power images showing colocalization of ChR2:eYFP (green) with ChAT (red) in the PBG of ChAT-Cre (***E***, top left) and VGLUT2-Cre (***F***, top right) mice. Bottom, ChR2:eYFP-positive terminals (green) in the main PBG projection targets SC, LGN, Opt, and LA of ChAT-Cre (***E***) and VGLUT2-Cre (***F***). Scale bar, 100 μm.

To determine whether ChAT-Cre or VGLUT2-Cre driver lines would robustly allow for cell-type-specific expression and colocalization of PBG cholinergic and glutamatergic neurons in adult animals, we injected Cre-dependent AAVs expressing ChR2:eYFP into the PBG of the respective lines (Fig. 4*A*). Injection of a Cre-dependent AAV will only label cells that express Cre recombinase at the time of or post-injection, but not cells that may have transiently expressed Cre only during development. Three weeks after AAV infusion, we performed immunohistochemistry to detect the overlap of VGLUT2-Cre driven ChR2:eYFP expression with ChAT protein, and found that, in accordance with our *in situ* data, 88.6 ± 2.88% (*n* = 4) of YFP^+^ (VGLUT2^+^) neurons colabeled with ChAT (Fig. 4*B*–*F*). Further, we analyzed expression of ChR2:eYFP in PBG terminals of both ChAT-Cre and VGLUT2-Cre injected animals to determine whether both lines labeled comparable terminal areas. We obtained strong labeling in both, ipsilateral and contralateral superior colliculi (SC), and primarily ipsilateral labeling of the lateral geniculate nucleus (LGN), optic tract (Opt), and lateral amygdala (LA) of both Cre driver lines. Additionally, we saw moderate labeling in the medial amygdala (MeA), but only weak labeling in the basolateral (BLA) or central (CeA) nuclei of the amygdala in both the lines.

**Figure 5.**
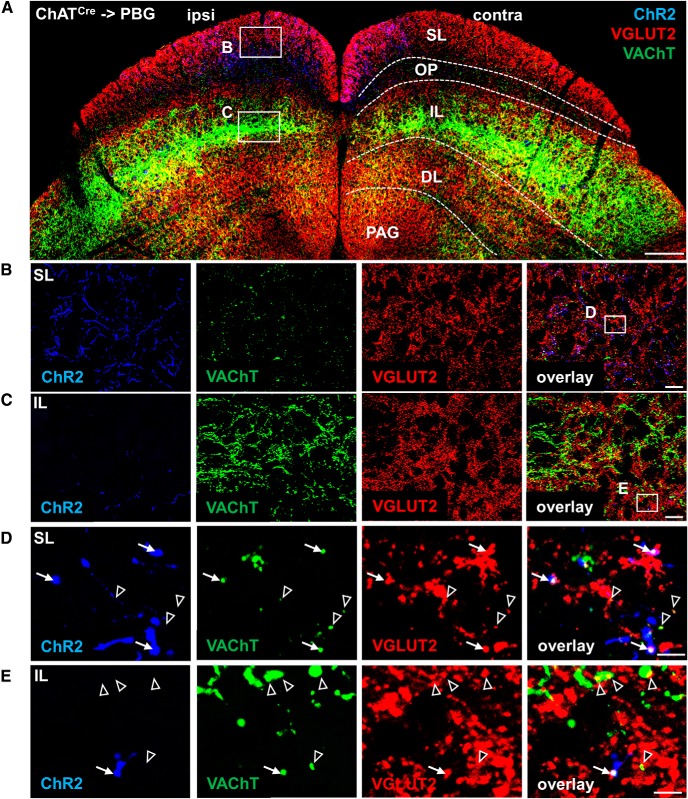
Partial colocalization of VGLUT2 and VAChT in PBG terminals in SC. ChAT-Cre mice injected with AAV5-DIO-ChR2:eYFP into the PBG were immunostained for ChR2 (blue), VGLUT2 (red), and VAChT (green). ***A***, Tiled SC image and overview of VGLUT2, VAChT, and ChR2 expression in SC sublayers; white squares labeled B and C indicate higher-magnification images shown in ***B*** and ***C***. PAG, Periaqueductal gray. Scale bar, 200 μm. ***B***, ***C***, High-power images demonstrating ChR2-labeled processes primarily in the SL (***B***) and sparser expression in the IL (***C***); white squares labeled D and E indicate zoomed-in images shown in ***D*** and ***E***. Scale bar, 20 μm. ***D***, ***E***, High-magnification images in SL (***D***) and IL (***E***) demonstrate partial colocalization of ChR2, VAChT, and VGLUT2 (indicated by white arrows) and partial colocalization of VAChT and VGLUT2 in ChR2-negative terminals (indicated by black/white arrows). Scale bar, 5 μm.

To determine whether VGLUT2 and the vesicular acetylcholine transporter (VAChT) can colocalize to the same terminals, we stained SC sections from ChAT-Cre mice injected with AAV5-DIO-ChR2:eYFP in the PBG with antibodies against VAChT and VGLUT2 (Fig. 5). VGLUT2 expression was rather homogenously distributed in all SC layers (with the weakest expression in the optic nerve layer), presumably reflecting multiple diverse VGLUT2^+^ inputs to SC. The bulk of VAChT signal was concentrated in the intermediate layer (IL), more moderate expression was seen in the deep layer (DL), and scant punctate expression was observed in the superficial layer (SL; Fig. 5*A*). In contrast, ChR2:eYFP labeled PBG terminals were densest in the ipsilateral and contralateral SL (Fig. 5*A*, but compare Fig. 4*E*) suggesting that PBG cholinergic neurons primarily target the SL, in line with previous reports (Graybiel, 1978; Usunoff et al., 2007). High-magnification imaging revealed that VAChT and VGLUT2 partially colocalized in ChR2:eYFP-labeled processes in both SL and IL (Fig. 5*B*–*E*). This suggests that VAChT and VGLUT2 can colocalize to the same SC terminals, but can distribute to separate terminals as well.

### Viral tagging reveals few PPTg glutamate neurons are cholinergic in the adult, but that PPTg glutamate and cholinergic neurons project to similar structures

**Figure 6.**
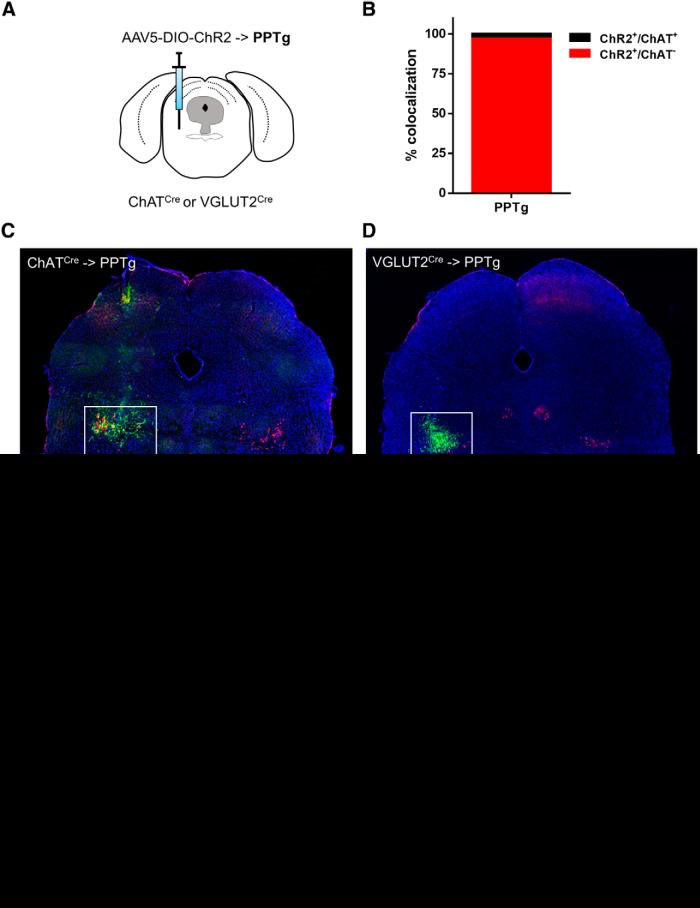
Viral tracing in PPTg neurons of ChAT-Cre and VGLUT2-Cre mice. ***A***, Schematic of AAV-DIO-ChR2 injection into the PPTg. ***B***, Quantification of ChR2+ cells co-positive for ChAT counted in the PPTg of VGLUT2-Cre mice; *n* = 6 animals. ***C***, ***D***, Tiled midbrain images of ChR2 (green) expression in the PPTg of ChAT-Cre (***C***) and VGLUT2-Cre (***D***) mice stained for ChAT (red) and counterstained with DAPI (blue). ***E***, ***F***, Higher-power images showing colocalization of ChR2 (green) with ChAT (red) in the PPTg of ChAT-Cre (***E***, top left) and VGLUT2-Cre (***F***, top right ) mice. ChR2-positive terminals (green) in the selected major PPTg projection targets VTA, SNc, GPi, LH, STN, and CeA/BLA of ChAT-Cre (***E***, bottom left ) and VGLUT2-Cre (***F***, bottom right ). Scale bar, 100 μm.

Injection of Cre-dependent AAV-DIO-ChR2-mCherry into the PPTg of VGLUT2-Cre mice (Fig. 6*A*), led to only a small percentage (3.0 ± 0.63%, *n* = 6) of overlap between ChR2:mCherry (VGLUT2^+^) and ChAT (Fig. 6*B*–*F*). This is less than what we found by *in situ* hybridization, but consistent with only a minor fraction of cholinergic PPTg neurons being co-positive for VGLUT2 in the adult. Nevertheless, tracing of ChR2-labeled processes revealed that cholinergic and glutamatergic neurons in the PPTg projected to overlapping terminal structures (Fig. 6*F*). We obtained strong fluorescent labeling in the ventral tegmental area (VTA), substantia nigra *pars compacta* (SNc), globus pallidus internal segment (GPi), lateral hypothalamus, and subthalamic nucleus (STN) of both, ChAT-Cre and VGLUT2-Cre mice (Fig. 6*F*). In contrast, we found that only cholinergic (ChAT-Cre), but not glutamatergic (VGLUT2-Cre) labeled terminals were present in the CeA and BLA suggesting that the amygdala is innervated selectively by cholinergic but not glutamatergic efferents from PPTg (Fig. 6*F*).

## Discussion

Accumulating evidence shows that many neuronal populations co-release or have the potential to co-release multiple of the classical neurotransmitters. The molecular identification of vesicular transporters for acetylcholine, monoamines, and GABA/glycine in the 1990s, and for glutamate in the early 2000s, led to the discovery of multiple vesicular neurotransmitter transporters in the same cells (El Mestikawy et al., 2011; Hnasko and Edwards, 2012). Because of the ubiquitous presence of the amino acid glutamate in cells, VGLUT expression has been shown both necessary and sufficient for the release of glutamate from multiple neuronal populations (Takamori et al., 2000; Featherstone, 2010; Steinkellner et al., 2018). Thus, the simple presence of VGLUTs establishes the potential for glutamate release. In contrast, storage and release of monoamines, ACh or GABA requires at least one or several other proteins to catalyze their biosynthesis or extracellular retrieval.

Central cholinergic neurons are widely and diffusely distributed throughout the forebrain, midbrain, hindbrain, and spinal cord, and many subpopulations of cholinergic neurons have been shown to express either VGLUT1 (MHb), VGLUT2 (spinal motor neurons), VGLUT3 (striatal cholinergic interneurons), or VGAT (forebrain; Gras et al., 2002; Herzog et al., 2004; Kraus et al., 2004; Nishimaru et al., 2005; Ren et al., 2011; Frahm et al., 2015; Saunders et al., 2015a,b). The expression of these vesicular transporters permits cholinergic cells to engage in glutamate or GABA-mediated fast excitatory or inhibitory neurotransmission. Co-released, glutamate and GABA can also modulate neurotransmission through actions on presynaptic and postsynaptic metabotropic receptors (Fasano et al., 2017; Cai and Ford, 2018; Chuhma et al., 2018). Cotransmission thus allows for a broader range of postsynaptic effects, across a wider range of timescales, that could contribute to the modulation and adaptation of presynaptic and postsynaptic signaling mechanisms based on physiologic needs. For instance, brief stimulation of VGLUT1-expressing MHb cholinergic projections to the IPN produces mainly fast, excitatory, glutamate-mediated currents; whereas long, tetanic stimulation induces slower, nAChR-mediated currents (Ren et al., 2011). Similarly, activation of PPTg cholinergic terminals in the CeA transiently excites CeA neurons through glutamate (Aitta-Aho et al., 2018), whereas PPTg cholinergic terminals in the substantia nigra may co-release glutamate or GABA depending on the exact anatomic location (Xiao et al., 2016; Estakhr et al., 2017).

In addition to postsynaptic consequences mediated by a second transmitter, co-release can also modulate presynaptic function. The vesicular uptake of one neurotransmitter can increase the uptake and storage of another transmitter when present on the same synaptic vesicles, a phenomenon termed “vesicular synergy” (El Mestikawy et al., 2011). For instance, striatal cholinergic interneurons express VGLUT3 and the concomitant uptake of glutamate increases the vesicular storage of ACh (Gras et al., 2008). Indeed, loss of VGLUT3 eliminated the glutamatergic postsynatpic currents evoked by stimulating striatal cholinergic interneurons, but also most of the cholinergic response (Nelson et al., 2014). Similarly, glutamate filling via VGLUT1 in the MHb can increase uptake of ACh (Frahm et al., 2015) and a similar mechanism appears to occur in subsets of midbrain dopamine and serotonin neurons (Amilhon et al., 2010; Hnasko et al., 2010; Aguilar et al., 2017).

In our study we focused on two essential mesopontine cholinergic nuclei: the PBG and the PPTg. The PBG is a small bilateral nucleus located in the dorsolateral midbrain and is thought to be a substructure of the SC (Cui and Malpeli, 2003; Usunoff et al., 2007). It mainly contains densely populated, small cholinergic neurons. We found that most PBG cholinergic neurons expressed VGLUT2 in the adult, and that VGLUT2 and VAChT partially colocalized in SC terminals, positioning these cells to co-release ACh and glutamate. The PBG is an important nucleus in the control of visuomotor behavior (Cui and Malpeli, 2003), and thus fast glutamate-mediated transmission may be required to rapidly adapt to changing visual cues in the environment. Further, PBG projections to the amygdala have been demonstrated to be crucially involved in fear responses in mice (Shang et al., 2015), and thus position the PBG as a key structure in the fast and early detection of visually-triggered fear responses.

Ventromedial to the PBG, the PPTg is located just caudal to the dopaminergic retrorubral field (A8 area) and contains large cholinergic neurons, as well as glutamate and GABA neurons (Wang and Morales, 2009; Mena-Segovia and Bolam, 2017; Yoo et al., 2017). Although our genetic fate-mapping approach suggested that most PPTg neurons were positive for VGLUT2, only a small subpopulation continued to express detectable VGLUT2 mRNA in the adult, and even fewer cholinergic neurons coexpressed VGAT mRNA. The fact that PPTg cholinergic neurons largely expressed the VGLUT2 reporter zsGreen suggests that these cells transiently expressed VGLUT2 during development, but largely repressed VGLUT2 later in life. However, the cholinergic and glutamatergic neurons in the PPTg displayed overlapping efferent projections suggesting that they may have common neuronal precursors or are involved in similar neuronal modulations. This is reminiscent of midbrain dopamine neurons: most of which are positive for VGLUT2 reporter in a fate-mapping experiment, but only a minority express detectable VGLUT2 mRNA in the adult, but neuronal injury led to VGLUT2 upregulation (Steinkellner et al., 2018). It is thus tempting to speculate that VGLUT2 might re-emerge in these cells under conditions of neuronal stress or injury such as in Parkinson’s or Alzheimer’s diseases.

Our results highlight the diversity and complexity of mesopontine cholinergic neurons, and suggest that PBG neurons which coexpress ChAT and VGLUT2 have the potential to engage in multiple modes of neurotransmission, whereas glutamate release from other cholinergic populations may be under dynamic developmental control.

*Note Added in Proof:* We refer interested readers to an article with overlapping conclusions that we became aware of while in press: Nasirova et al., 2019. Dual recombinase fate mapping reveals a transient cholinergic phenotype in multiple populations of developing glutamatergic neurons, Journal of Comparative Neurology, in press.

## Synthesis

Reviewing Editor: Quentin Pittman, University of Calgary Decisions are customarily a result of the Reviewing Editor and the peer reviewers coming together and discussing their recommendations until a consensus is reached. When revisions are invited, a fact-based synthesis statement explaining their decision and outlining what is needed to prepare a revision will be listed below. The following reviewer(s) agreed to reveal their identity: Salah El Mestikawy, Juan Mena-Segovia.

Using a genetic fate-mapping strategy the authors found that a large majority (>90%) of cholinergic mesopontine neurons (in mice) express VGLUT2 at some point of their development (except the oculomotor nucleus (3N)). In the adult only the parabigeminal nucleus (PBG) and some pedunculopontine tegmental nucleus (PPTG) neurons maintain this expression. In comparison, only a limited number of these neurons (2-5%) developmentally expressed the vesicular GABA transporter (VGAT). The authors also injected AAV expressing flex fluorescent tag (combined to chanel rodhopsin) in the PBG and PPTg to label projection area of these neurons.

This is a well conducted and solid anatomical study using a combination of genetic and sensitive in situ method as well AAV injection to trace projection pathways. It is well written and reader friendly. Information provided by this study will be useful for those working on mesopontine cholinergic neurons as well as for those using VGLUT2-CRE mouse line.

Major comments

1) One important question that is not addressed in this study is whether VAChT and VGLUT2 expressed by adult PBG neurons are sent to the same or to segregated terminals. This question could be addressed by performing triple labeling with sections from PBG injected CHAT-CRE mice (VGLUT2/VAChT/CHR2-YFP). You most likely already possess the material of the projections of the PBG neurons and these data could nicely complement Figure 4.

Minor points

2) Page 1 line 19-20: in the abstract “support a growing body of literature indicating that distinct cholinergic populations have differing potential for neurotransmitter co-release.”

To my knowledge this applies only to Ach and GABA or glutamate co-release. This should be specified.

3) Page 1-2, lines 23-29 sounds more like Introduction than significance statement

4) Page 2 line 38: “neurotransmission is mediated by a surprisingly small group of recycling transmitters”.

I am disturbed by the term “recycling transmitters” or “classical recycling neurotransmitters”. If this refers to reuptake of neurotransmitters, then ACh is not “recycled” but degraded, resynthesized and then only reused. The term “classical” (although not perfect either) could be used?

5) Page 2 line 48-49:

- concerning Ach and glutamate co-transmission add reference Sakae et al. 2015 (PMID: 21315256).

- concerning glutamate and 5-HT co-transmission add reference Sengupta et al. 2017 (PMID: 28087766)

- The vesicular “GABA” transporter also translocate glycine. Therefore the name VIAAT (vesicular inhibitory amino acid transporter) is more accurate.

6) Were Slc17a6IRESCre mice validated in this brain area ? If yes how ? I am surprised by the relatively limited number of VGUT2-positive neurons on Figure 2C.

7) Page 7 line 158: “with mice expressing Cre recombinase driven by VGLUT2 regulatory elements”

This sentence is a bit misleading. With this type of construct, the CRE gene is inserted after an IRES signal positioned after the stop codon of VGLUT2 last coding exon. IRES allows for translation initiation of a second gene (in this case). The terms “VGLUT2 regulatory elements” suggest that the VGLUT2 promoter was use to drive CRE expression.

8) Figure 3C would it be possible to indicate the % of VGLUT2-only and VGAT-only neurons in cholinergic mesopontine nuclei (even though according to the figure their number seem ultra-low) ?

9) Page 11 line 255-257: “The expression of these vesicular transporters thus suggests the potential of these cells to engage in fast excitatory or inhibitory neurotransmission in addition to ACh-mediated volume transmission.”

Caution, this is only partially true. In the case of glutamate, it has been found that glutamate released by hippocampal GABAergic interneurons can activate metabotropic receptor (Fasano et al, 2017, PMID: 25798091).

10) Fate mapping and in situ hybridization were provided for PBG, PPTg and LDT, but virus injections were only reported for PBG and PPTg. Having the complete dataset for the LDT would be valuable if this were available.

11) Was the in situ hybridization performed in wild-type mice?

12) There are a few mentions about the likelihood of acetylcholine being released by volume transmission from PPTg axons, but this claim is unsubstantiated and outdated, particularly in light of recent optogenetic studies as well as those demonstrating synapses formed by PPTg cholinergic axons on their target structures.
